# Characteristics of HER2-negative breast cancers with FISH-equivocal status according to 2018 ASCO/CAP guideline

**DOI:** 10.1186/s13000-021-01187-z

**Published:** 2022-01-07

**Authors:** Hui Kong, Qianming Bai, Anqi Li, Xiaoyan Zhou, Wentao Yang

**Affiliations:** 1 Department of Pathology, Fudan University Shanghai Cancer Center, Shanghai, 200032 China; 2 Department of Pathology, Zhongshan Hospital, Fudan University, Shanghai, 200032 China

**Keywords:** Breast cancer, HER2 status, 2018 ASCO/CAP

## Abstract

**Background:**

According to 2018 ASCO/CAP guideline, HER2 FISH-equivocal breast cancers will be categorized as HER2 negative except those with IHC 3+. However, whether or not HER2 FISH-equivocal breast cancers was a heterogeneous group has not been well illustrated.

**Methods:**

195 HER2 FISH-equivocal breast cancer samples were collected from 2014 to 2018. The molecular subtype was identified according to 2013 St Gallen consensus, and HER2 status was also re-determined following 2018 ASCO/CAP guideline. All samples were classified into 4 groups according to the average *HER2* copy number (4.0–4.4, 4.5–4.9, 5.0–5.4, 5.5–5.9 signals/cell). The relationship between *HER2* copy number and clinicopathological parameters was analyzed.

**Results:**

183 (93.8%) of 195 FISH-equivocal cases were classified as luminal-like subtype, while the other 12 (6.2%) were undetermined. Following 2018 ASCO/CAP guideline, all FISH-equivocal cases were recategorized as HER2 negative. Therefore, 31(15.9%) cases were luminal A-like, 152 (77.9%) were luminal B-like (HER2 negative) and 12 (6.2%) were triple negative. The average *HER2* copy number showed a positive correlation with chromosome 17 polysomy, but had no significant association with other clinicopathological parameters as well as prognosis. 17 (8.7%) patients were treated with trastuzumab, but showed no difference in prognosis with those who didn’t receive targeted therapy.

**Conclusions:**

In this study, all HER2 FISH-equivocal breast cancers were recategorized as HER2 negative according to 2018 ASCO/CAP guideline. Most of these patients were luminal B-like (HER2 negative). The average HER2 copy number had no significant association with clinicopathological parameters, as well as prognosis.

**Supplementary Information:**

The online version contains supplementary material available at 10.1186/s13000-021-01187-z.

## Background

Breast cancer remains the most frequently diagnosed cancer and the leading cause of cancer death among females worldwide. According to global cancer statistics 2018, there were around 2.1 million new cases, accounting for almost 25% of all female cancers [1].

Human epidermal growth factor receptor 2 (HER2) is involved in the regulations of several key cellular signal pathways in breast cancers, including proliferation, migration, and adhesion [2]. HER2-targeted drugs, such as Trastuzumab, which inhibit downstream signaling of these pathways, are effective for HER2 positive breast cancer patients. Therefore, HER2 status is crucial for selection of treatment options. Currently, HER2 amplification status is mainly determined by immunohistochemistry (IHC) assays for protein overexpression and fluorescence in situ hybridization (FISH) for gene amplification. Typically, IHC assays are the first adopted method, *HER2* FISH is required when the IHC result is equivocal.

HER2 status was determined according to the American Society of Clinical Oncology/College of American Pathologists (ASCO/CAP) guideline [3–5]. However, 2013 ASCO/CAP guideline caused an increased HER2 equivocal cases [6–8], whose HER2 status was not clear. It was hard for medical oncologists to decide whether to use HER2-targeted therapy for equivocal cases. Fortunately, the guideline was updated in 2018 and recommended a definitive diagnosis for the former *HER2* FISH-equivocal breast cancers. Almost all equivocal cases were categorized as HER2 negative except those with HER2 IHC 3+ [5]. Since the dual-probe FISH test provides an exact score of average *HER2* copy number, which ranges from 4.0 to 6.0, whether or not *HER2* FISH-equivocal breast cancers could be heterogeneous groups with regard to *HER2* copy number has not been well investigated.

In this study, we collected 195 *HER2* FISH-equivocal invasive breast cancers diagnosed following 2013 ASCO/CAP guideline [4], and re-determined the HER2 status according to 2018 updated guideline. The molecular subtype of those cases were identified based on HER2 status. We analyzed the relationship between *HER2* copy number and clinicopathological parameters. The impact of different *HER2* copy number on prognosis was also investigated.

## Methods

### Patients and study design

195 breast cancers samples, which were considered *HER2* FISH-equivocal according to 2013 ASCO/CAP guideline, were collected from Shanghai Cancer Center from 2014 to 2018. Both IHC and FISH assays were performed on all the cases. The diagnoses were reviewed by two experienced senior pathologists. Clinical characteristics of these 195 study subjects are showed in Table 1.

**Table 1:** Characteristics of study subjects

	Variable	n (%)
**Age, yr**	**Median (range)**	53 (28–82)
**Histologic subtype**		
	**Ductal, NOS**	181 (92.8)
	**Lobular**	4 (2.1)
	**Micropapillary**	9 (4.6)
	**Solid papillary with invasion**	1 (0.5)
**Histologic grade**		
**I**		2 (1.0)
**II**		94 (48.2)
**III**		99 (50.8)
**ER status**		
**ER+**		183 (93.8)
**ER-**		12 (6.2)
**PR status**		
**PR+**		160 (82.1)
**PR-**		35 (17.9)
**HER2 status**		
**IHC status**		
**1+**		10 (5.1)
**2+**		185 (94.9)
**Dual-probe ISH status**		
**±**		195 (100.0)
**Polysomy 17**		
**Yes**		137 (70.3)
**No**		58 (29.7)
**Ki-67**		
**<20%**		49 (25.1)
**≥ 20%**		146 (74.9)
**TNM stage**		
**T (Primary tumour)**		
**T1**		93 (47.7)
**T2**		98 (50.3)
**T3**		1 (0.5)
**T4**		3 (1.5)
**N (Regional lymph nodes)**		
**N0**		104 (53.3)
**N1**		57 (29.2)
**N2**		19 (9.8)
**N3**		15 (7.7)
**M (Distant metastasis)**		
**M0**		192 (98.5)
**M1**		3 (1.5)
**Treatment**		
**Chemotherapy**		159 (81.5)
**Radiotherapy**		89 (45.6)
**Hormonal therapy**		123 (63.1)
**Trastuzumab**		17 (8.7)

HER2 status was re-determined following 2018 ASCO/CAP guideline. Molecular subtype was identified according to the 2013 St Gallen consensus [9]. We classified the 195 cases into 4 groups according to the average *HER2* copy number (4.0–4.4, 4.5–4.9, 5.0–5.4, 5.5–5.9 signals/cell, respectively). Histological subtype, grade, ER staus, PR status and chromosome 17 polysomy were investigated among 4 groups to find out the association between these clinicopathological factors with *HER2* copy number.

### IHC and FISH

IHC staining was performed using the Ventana Bench Mark ultra autostainer and the Ventana Ultra View universal DAB detection kit (Ventana Medical System Inc., Roche Tucson, Arizona, USA). The primary antibodies including ER, PR, HER2, Ki-67, E-cadherin, p120, AE1/AE3, calponin and p63 were purchased from Roche Ventana. All IHC stains were carried out with appropriate positive and negative controls.

The status of ER and PR were determined following ASCO/CAP guideline [10]. The ratio of nuclei-positive tumor cells to all tumor cells represented Ki-67 expression level and a Ki-67 expression was high when the ratio was ≥20% [9]. E-cadherin and p120 were employed to discriminate lobular carcinomas from ductal carcinomas. Double immunostainings were performed with AE1/AE3/P63 or AE1/AE3/calponin to confirm the presence of an invasive component.

A dual-probe FISH test using the PathVysion HER2 DNA probe Kit (Vysis Inc., Downers Grove, IL) was performed on the same specimen as IHC test following the manufacturer’s instructions. FISH results were interpreted by two experienced pathologists independently.

### Follow-up information

Long-term follow-up were carried out in Shanghai Cancer Center. The overall survival (OS) was defined as the time from the date of surgery to death from any cause. The Disease-free survival (DFS) was defined as the time from the date of surgery to the first observed event including recurrence, distant metastasis, second primary tumor and death of any cause. The last follow-up was conducted in August 2018.

### Statistical analysis

The correlation between clinicopathological factors and the average *HER2* copy number was analyzed by Chi-squared test or Fisher’s exact test. OS and DFS were described by Kaplan-Meier curves. IBM SPSS Statistics software (version 21) was used to perform the statistical analysis. All *P* values were two-tailed, and *P* value < 0.05 was considered to be statistically significant.

## Results

### Histologic subtype and grade

With regard to histologic subtype, 181 (92.8%) of the 195 *HER2* FISH-equivocal cases were invasive ductal carcinoma. 4 (2.1%) cases were invasive lobular carcinoma, and 9 (4.6%) cases were invasive micropapillary carcinoma, and only 1 (0.5%) case was solid papillary with invasion.

With regard to histological grade, the collected samples were distributed mainly between Grade II and Grade III. The numbers of corresponding cases were 94 (48.2%) and 99 (50.8%), respectively. Only 2 (1%) cases were diagnosed as Grade I.

### HER2 status

IHC and dual-probe FISH assays tests were performed to determine HER2 status. Among the 195 patients screened for the study, HER2 expression by IHC was as follows: IHC 1+ in 10 (5.1%), IHC 2+ in 185(26%) and IHC 3+ in 0 (0%). In other words, 10 cases were negative and the rest were equivocal in terms of HER2 status.

Dual-probe FISH analysis showed that 195 (100%) cases were FISH equivocal according to 2013 ASCO/CAP guideline. All the cases were recategorized as HER2 negative according to 2018 updated ASCO/CAP guideline.

### Molecular subtype

In this study, the molecular subtype was identified in accordance with 2013 St Gallen International Expert consensus, which mainly took the expressions of ER, PR, HER2 and Ki-67 as indicators.

The ratios of ER positive and PR positive were 93.8% (183/195) and 82.1% (160/195) respectively. The Ki-67 expression of 146 (74.9%) cases was high, and the rest 49 cases (25.1%) were low, when the cutoff value was set to be 20%. The HER2 status of samples differ according to 2013 or 2018 ASCO/CAP guidelines as described previously.

**Fig. 1:**
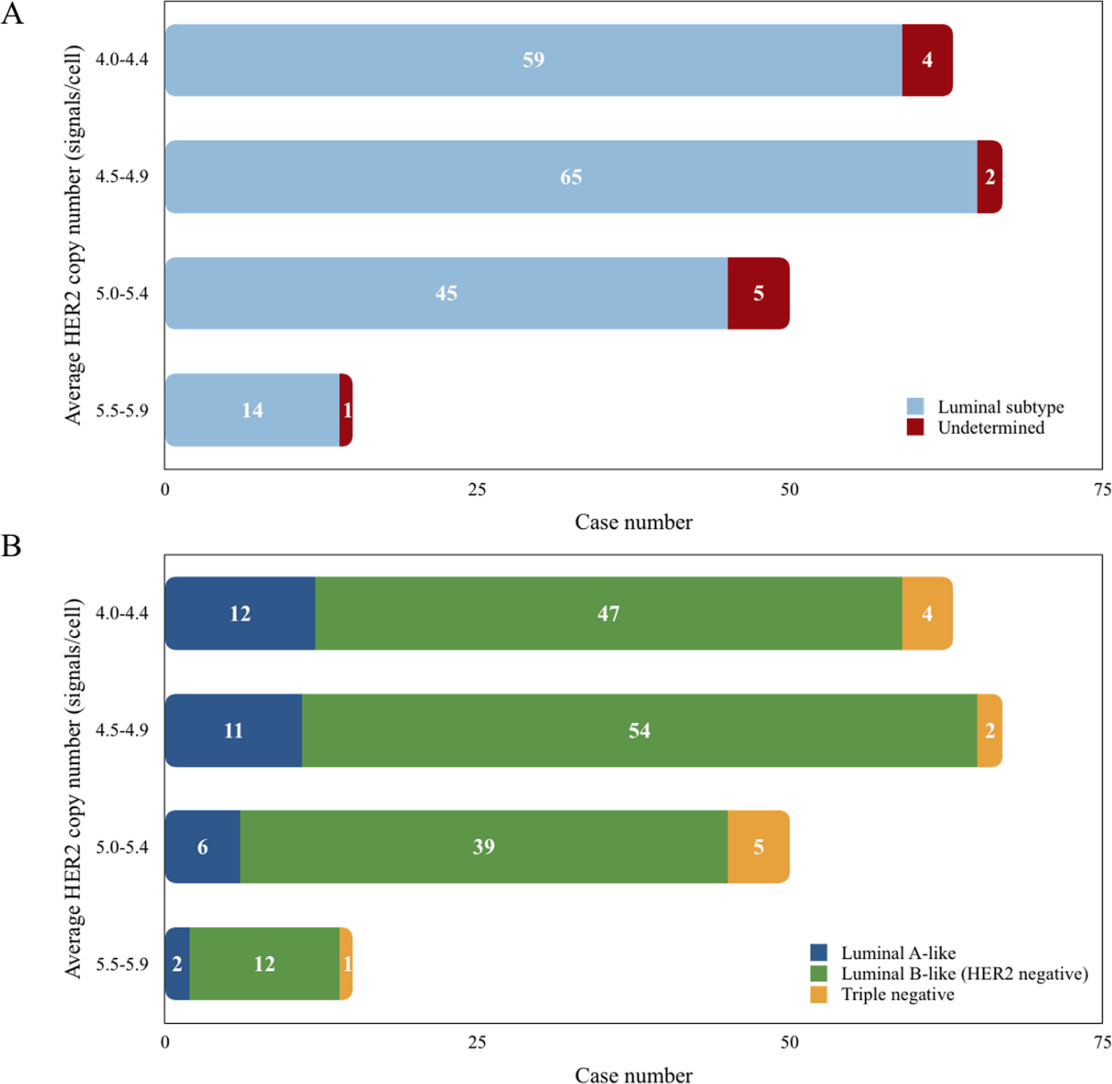
Molecular subtype distribution of HER2 FISH-equivocal breast cancers in terms of average HER2 copy number according to 2013 (A) and 2018 (B) ASCO/CAP guideline

In this study, 195 *HER2* FISH-equivocal samples were classified into 4 groups (A, B, C, D) according to the average *HER2* copy number (4.0–4.4, 4.5–4.9, 5.0–5.4, 5.5–5.9 signals/cell, respectively). The number of cases in each group were 63, 67, 50 and 15, respectively (Table 2). As shown in Fig. 1, there were 59 luminal subtype and 4 undetermined subtype breast cancer in group A, 65 luminal subtype and 2 undetermined subtype breast cancer in group B, 45 luminal subtype and 5 undetermined subtype breast cancer in group C, 14 luminal subtype and 1 undetermined subtype breast cancer in group D. Within 195 *HER2* FISH-equivocal patients, there were183 (93.8%) luminal subtype and 12 (6.2%) undetermined subtype breast cancer according to 2013 ASCO/CAP guideline. After HER2 status was re-determined following 2018 ASCO/CAP guideline, all the undetermined subtype cases became triple negative. The luminal subtype could be further classified into luminal A-like and luminal B-like (HER2 negative). In summary, 31(15.9%) cases were luminal A-like, and 152 (77.9%) were luminal B-like (HER2 negative) and 12 (6.2%) were triple negative.

**Table 2:** Correlations of average *HER2* copy number and major clinicopathological factors

Characteristics	Average ***HER2*** copy number				***P*** value
	4.0–4.4	4.5–4.9	5.0–5.4	5.5–5.9	
**Age (year)**					0.15
**≤ 53**	32 (50.8)	41 (61.2)	20 (40.0)	5 (33.3)	
**>53**	31 (49.2)	26 (38.8)	30 (60.0)	10 (66.7)	
**Histopathology**					0.969
**IDC**^a^	58 (92.1)	62 (92.5)	49 (98.0)	12 (80.0)	
Other IBC^b^	5 (7.9)	5 (7.5)	1 (2.0)	3 (20.0)	
**Histologic grade**					0.129
**I**	0 (0.0)	0 (0.0)	2 (4.0)	0 (0.0)	
**II**	27 (42.9)	34 (50.7)	23 (46.0)	10 (66.7)	
**III**	36 (57.1)	33 (49.3)	25 (50.0)	5 (33.3)	
**ER**					0.538
**Positive**	59 (93.7)	65 (97.0)	45 (90.0)	14 (93.3)	
**Negative**	4 (6.3)	2 (3.0)	5 (10.0)	1 (6.7)	
**PR**					0.257
**Positive**	48 (76.2)	57 (85.1)	43 (86.0)	12 (80.0)	
**Negative**	15 (23.8)	10 (14.9)	7 (14.0)	3 (20.0)	
**Ki-67**					0.065
**< 20%**	19 (30.2)	20 (29.9)	7 (14.0)	3 (20.0)	
**≥ 20%**	44 (69.8)	47 (70.1)	43 (86.0)	12 (80.0)	
**Polysomy 17**					<0.001**
**Yes**	30 (47.6)	48 (71.6)	46 (92.0)	13 (86.7)	
**No**	33 (52.4)	19 (28.4)	4 (8.0)	2 (13.3)	
**Primary Tumor (T)**					0.776
**T1**	31 (49.2)	33 (49.3)	21 (42.0)	8 (53.3)	
**T2**	30 (47.6)	33 (49.3)	28 (56.0)	7 (46.7)	
**T3**	0 (0.0)	1 (1.5)	0 (0.0)	0 (0.0)	
**T4**	2 (3.2)	0 (0.0)	1 (2.0)	0 (0.0)	
**Regional Lymph Nodes (N)**					0.806
**N0**	32 (50.8)	40 (59.7)	25 (50.0)	7 (46.7)	
**N1**	20 (31.7)	15 (22.4)	17 (34.0)	5 (33.3)	
**N2**	8 (12.7)	6 (9.0)	3 (6.0)	2 (13.3)	
**N3**	3 (4.8)	6 (9.0)	5 (10.0)	1 (6.7)	
**Distant Metastasis (M)**					0.804
**M0**	62 (98.4)	66 (98.5)	50 (100.0)	14 (93.3)	
**M1**	1 (1.6)	1 (1.5)	0 (0.0)	1 (6.7)	

^a^Invasive ductal cancer

^b^other types of invasive breast carcinomas (IBC), including invasive lobular carcinoma, invasive micropapillary carcinoma, and solid papillary carcinoma with invasion

### Correlation with Clinicopathological parameters

The principal clinicopathological parameters investigated included age, histological subtype, histological grade, ER status, PR status, and HER2 status, the presence of chromosome 17 polysomy, Ki-67 status and TNM stage.

The correlation between those parameters and average *HER2* copy number were analyzed. The results suggested that the average *HER2* copy number showed positive correlation with the presence of chromosome 17 polysomy (*P* < 0.001), but had no significant correlation with other clinicopathological parameters.

### Prognosis

The individualized therapy regimen was decided at a multidisciplinary team meeting. All involved patients were taken to surgery and treated with different combinations of chemotherapy, radiotherapy and hormonal therapy. In addition, within the 195 *HER2* FISH-equivocal patients, 17 (8.7%) received trastuzumab targeted therapy preoperatively or postoperatively.

Patient follow-up data was collected in a long term and the most recent data was collected in August 2018. Among the 195 study subjects, 16 patients loss of contact were excluded from survival analysis. The median duration of follow-up was 34 months (range 10–55). By the end of data collection, no patients experienced recurrence of breast cancer, while 5 patients had distant metastasis, including one lung metastasis, one liver metastasis, and three bone metastases. Besides, 4 patients passed away without detail information.

Treatment response and prognosis of 17 patients receiving HER2 targeted therapy were the major problem concerned. Two of them were treated with trastuzumab before surgery, and they also received chemotherapy and/or radiotherapy. The Miller-Payne grading system was used to assess pathologic response to neoadjuvant chemotherapy [11], and the two patients were categorized as grade 3 and grade 4. The other 15 patients took targeted therapy after surgery, and nobody exprienced recurrence by the end of the follow-up.

Univariate analysis was performed to reveal the impact of prognostic factors on DFS and OS of breast cancer patients. The results showed that the N (regional lymph nodes) stage significantly affected OS, and the M (distant metastasis) stage significantly affected both DFS and OS. However, the average *HER2* copy number in FISH test had no significant impact on DFS and OS. Meanwhile, DFS and OS showed no significant difference between patients with and without targeted therapy (Table 3).

**Table 3:** Univariate analysis of prognostic factors affecting DFS and OS in HER2 FISH-equivocal cancers

Factors	Subset	DFS	OS
		***P*** value	***P*** value
**Age (year)**	≤ 53 />53	0.299	0.059
**Histologic subtype**	IDC / Other IBC	0.917	0.978
**Histologic grade**	I / II / III	0.888	0.967
**ER**	Positive / Negative	0.425	0.531
**PR**	Positive / Negative	0.539	0.146
**HER2 IHC score**	1+ / 2+	0.518	0.643
**Average HER2 copy number**	1 / 2 / 3 / 4^a^	0.897	0.887
**Ki-67**	< 20% / ≥ 20%	0.674	0.167
**Target therapy**	Yes / No	0.439	0.582
**T stage**	T1 / T2 / T3 / T4	0.965	NA
**N stage**	N0 / N1 / N2 / T3	0.146	0.009*
**M stage**	M0 / M1	0.003*	0.000*

^a^Four groups with regard to average HER2 copy number: 4.0–4.4, 4.5–4.9, 5.0–5.4, 5.5–5.9 signals/cell

## Discussion

HER2 amplification is constantly a poor prognostic factor of breast cancer. Fortunately, there are a number of HER2-targeted therapies available today, which markedly inhibits tumor growth and prolongs survival of HER2-positive breast cancer patients [12, 13]. Therefore, it is crucial to identify HER2 status before therapy.

Immunohistochemical analyses and FISH procedures are two approved methodologies to identify HER2 status of breast cancer specimens. HER2 status might be still ambiguous in some scenarios. The 2018 ASCO/CAP guideline recommended a rigorous diagnostic approach to identify HER2 status. According to the updated guideline, HER2 FISH-equivocal breast cancers will be categorized as HER2 negative except those with IHC 3+. In our study, 195 FISH equivocal breast cancers consisted of 10 (5.1%) IHC 1+ cases and 185 (94.9%) IHC 2+ cases.. Press et al. reported similar findings in their study that only one case among 134 carcinomas was HER2 IHC 3+ [14]. Therefore, all the 195 equivocal cases were recategorized as HER2 negative in our study.

The molecular subtype of breast cancers could help us to understand the different clinical characteristics, treatment response and prognosis. HER2 FISH-equivocal carcinomas without determined subtype in our study were classified into luminal B-like (HER2 negative) according to the updated guideline. Guo et al. also demonstrated that HER2 equivocal cases according to 2013 ASCO/CAP guideline had similar biological behavior with luminal B type tumors [6]. Besides, Tong et al. reported similar clinicopathological features and survival outcome between HER2 equivocal and negative cases in the absence of HER2 targeted therapy [15].

Evidence from previous clinical trial [16] showed that patients with HER2 equivocal and HER2 negative carcinomas experienced similar overall survivals and disease-free survivals. The Expert Panel of ASCO/CAP decided that HER2 FISH equivocal tumors with IHC 2+ or 1+ should no longer be treated with HER2-targeted therapy. In our study, 17 *HER2* FISH-equivocal individuals received targeted therapy preoperatively or postoperatively. However, the sample size was so small to preclude power enough for valid statistical analysis. Further evidence is required to conclude on whether this population would benefit from HER2-targeted therapy.

The average *HER2* copy number ranged from 4.0 to 6.0 in FISH equivocal tumors. However, no significant correlation was observed between average *HER2* copy number and clinicopathological factors, OS and DFS in our study. We found that chromosome 17 polysomy had positive correlation with average HER2 copy number. Actually, polysomy 17 is a major cause of HER2 FISH equivocal [17]. Generally, breast cancer patients with polysomy 17 have not been considered eligible for HER2 targeted therapy. Nevertheless, researches showed that complete chromosome 17 polysomy was rare. Patients with polysomy 17 have a high possibility to be HER2 positive with concomitant amplification of the centromeric region [18, 19]. Further investigation is still warranted to confirm whether polysomy 17 breast cancers could benefit from HER2-targeted therapy.

## Conclusions

In conclusion, our study revealed that all HER2 FISH-equivocal breast cancers were recategorized as HER2 negative according to 2018 ASCO/CAP guideline. Moreover, Most of these patients were luminal B-like (HER2 negative). The average HER2 copy number was positively correlated with the presence of chromosome 17 polysomy, but showed no significant correlation with other clinicopathological factors, such as histological subtype, histological grade, ER and PR status. Patient survival and tumor response to HER2-targeted therapy remains further investigation .

## Supplementary Information

**Additional file 1 Fig. S1 A.** Invasive lobular carcinoma, classic type. **B.** Immunohistochemistry for E-cadherin showing an absence of membranous staining in tumors cells. **C.** Immunohistochemistry for p120-catenin showing cytoplasmic staining in the tumor cells. **D.** Invasive micropapillary carcinoma. **E.** Immunohistochemistry for EMA (MUC1) showing the inside-out growth pattern. **F.** Solid papillary carcinoma with invasion.

## Data Availability

The datasets used and/or analyzed in the this study are available from the corresponding author on reasonable request.

## Abbreviations

ASCO/CAP: American Society of Clinical Oncology/College of American Pathologists
HER2: Human epidermal growth factor receptor 2
FISH: Fluorescence in situ hybridization
IHC: Immunohistochemistry
OS: Overall survival
DFS: Disease-free survival
