# Supplementary figures and images for: Characteristics of HER2-negative breast cancers with FISH-equivocal status according to 2018 ASCO/CAP guideline
Source: Diagn Pathol. 2022 Jan 7;17:5. doi: 10.1186/s13000-021-01187-z (PMC8742337; doi:10.1186/s13000-021-01187-z)

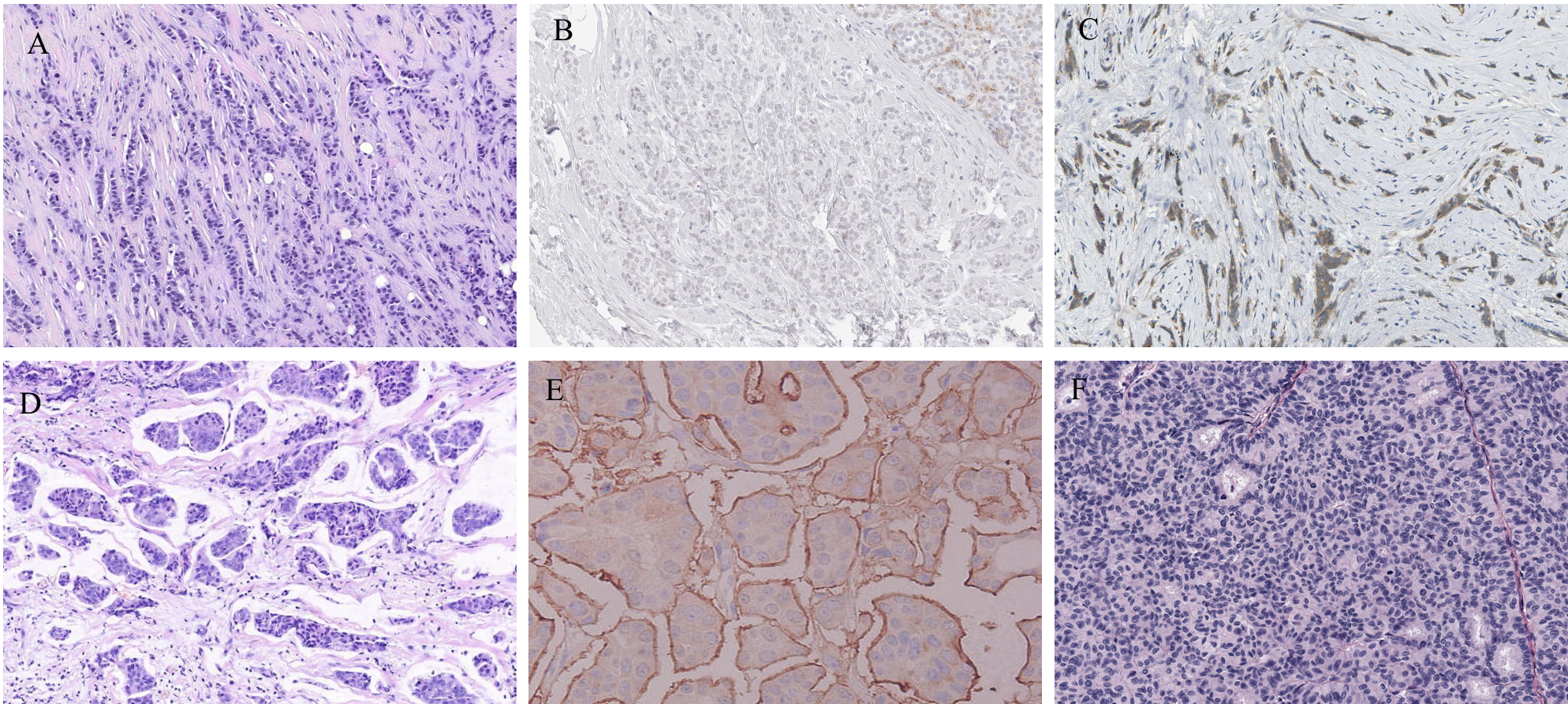

Supplement: Supplementary file 1 — Additional file 1 Fig. S1 A. Invasive lobular carcinoma, classic type. B. Immunohistochemistry for E-cadherin showing an absence of membranous staining in tumors cells. C. Immunohistochemistry for p120-catenin showing cytoplasmic staining in the tumor cells. D. Invasive micropapillary carcinoma. E. Immunohistochemistry for EMA (MUC1) showing the inside-out growth pattern. F. Solid papillary carcinoma with invasion. [file 13000_2021_1187_MOESM1_ESM.tiff]
